# Related but different: distinguishing postpartum depression and fatigue among women seeking help for unsettled infant behaviours

**DOI:** 10.1186/s12888-018-1892-7

**Published:** 2018-09-25

**Authors:** Nathan Wilson, Karen Wynter, Jane Fisher, Bei Bei

**Affiliations:** 1 Monash Institute of Cognitive and Clinical Neurosciences, School of Psychological Sciences, Monash University, 18 Innovation Walk, Clayton Campus, VIC 3800 Australia; 2 Global Public Health Unit, School of Public Health and Preventative Medicine, Monash University, Clayton, Victoria Australia; 3 Centre for Quality and Patient Safety Research – Western Health Partnership, School of Nursing and Midwifery, Faculty of Health, Deakin University, Burwood, VIC Australia; 4 Masada Early Parenting Centre, Masada Private Hospital, East St Kilda, VIC Australia

**Keywords:** Postpartum, Depression, Fatigue, Postnatal, Confirmatory factor analysis, Depressive

## Abstract

**Background:**

A growing body of evidence in relatively healthy populations suggests that postpartum depression and fatigue are likely distinct but related experiences. However, differentiating depression and fatigue in clinical settings remains a challenge. This study aimed to assess if depression and fatigue are distinct constructs in women with relatively high fatigue and psychological distress symptoms attending a residential program that assists with unsettled infant behaviour.

**Methods:**

167 women (age: *M* = 34.26, *SD* = 4.23) attending a private residential early parenting program completed the Depression Anxiety Stress Scale (DASS21-D), Fatigue Severity Scale (FSS) and self-report sleep variables before program commencement. Confirmatory Factor Analysis examined the associations between depression and fatigue latent factors.

**Results:**

A two-factor model of separate but related depression and fatigue constructs provided a significantly better fit to the data than a one-factor model of combined depression and fatigue (*p* < .001). In the two-factor model, the depression and fatigue latent factors were moderately correlated (.41). Further predictive utility of this two-factor model was demonstrated as both depression and fatigue factors were independently predicted by worse self-reported sleep efficiency.

**Conclusions:**

This study provides empirical evidence that for women attending a clinical service with relatively high fatigue and psychological distress, postpartum depression and fatigue remain separate but related experiences. These findings suggest that in women seeking clinical support in the postpartum period, both depression and fatigue need to be carefully assessed to ensure accurate diagnoses, and (b) whilst depression intervention may improve fatigue, targeted fatigue intervention may also be warranted.

**Electronic supplementary material:**

The online version of this article (10.1186/s12888-018-1892-7) contains supplementary material, which is available to authorized users.

## Background

Maternal depression and fatigue symptoms are both prevalent across the first two years after giving birth, with 10 to 20% reporting depressive symptoms and 40 to 60% reporting fatigue symptoms [1–3]. This may be at least partly due to the under-recognized nature of women’s caregiving work and the potential for occupational fatigue associated with the demands of infant caregiving [4]. Within this context, depression and fatigue can share complex bi-directional relationships. Fatigue is one of the most common symptoms of depression and part of the diagnostic criteria for depressive disorders [5, 6]. Several postpartum studies have reported significant positive univariate associations between depressive and fatigue symptoms within the first 32 weeks postpartum [7]. Depression and fatigue may also predict each other over time: across the first four years postpartum, depressive symptoms have been shown to predict future fatigue levels, and vice versa [8–10].

### Depression and fatigue in community samples of postpartum women

Given this close relationship between depression and fatigue, there has been a debate as whether they are distinct phenomena [11, 12]. In relatively healthy women in the postpartum period, evidence points to depression and fatigue being two different constructs [11, 13]. A qualitative study found that women with depressive symptoms reported symptoms such as feelings of emptiness and guilt that were not endorsed by non-depressed but fatigued women [14]. This is consistent with studies that identified clusters of women with high fatigue but not depressive symptoms [15, 16]. Two studies examined specific symptom constructs of postpartum depression and fatigue using confirmatory factor analysis (CFA) in community populations within the first year postpartum [11, 13] and one study also at four years postpartum [13]: both studies concluded that a two-factor model of related but separate latent factors of depression and fatigue provided a better fit to the data than a single combined factor at all time-points.

### What about women experiencing elevated postpartum fatigue and distress?

The differentiation of depression and fatigue symptoms has not been well examined in a clinical setting. Findings among healthy women may not generalise to those with elevated psychological distress and fatigue symptoms seeking clinical care. Depression and fatigue share many common features that can make them difficult to differentiate in clinical settings [14, 17]. For example, they may share similar indicators among women seeking clinical help, such as irritability, feeling overwhelmed, and impaired physical and cognitive functioning [13, 14, 18, 19]. Depression and fatigue can also share underlying causes such as sleep disturbance, physiological changes, or situational factors (e.g., unsettled infant behaviours; [12, 18, 20]). Together, these similarities in presentation and causes present a challenge in differentiating depression and fatigue and can lead to potential over-diagnosis of fatigue as depression [10, 13].

While there is evidence that fatigue and depression are related but separate constructs in healthy populations, it is possible that as depression and fatigue levels increase, they become less distinct and harder to differentiate [11, 17]. High fatigue symptoms may reduce self-care behaviours and pleasurable activities, which may contribute to low mood [11]. Conversely, it is also possible that distinct features of both depression and fatigue may become more apparent as symptom severity increases [11].

Better understanding of the relationship between depression and fatigue in mothers at risk for both conditions is of critical importance to clinical services for both assessment and treatment. It is currently routine practice in many postpartum settings to screen for depressive disorders, but the assessment of fatigue is not routine [6, 13]. If symptoms of fatigue and depression largely overlap, existing short screening measures of depressive symptoms may be sufficient, and treatments for postpartum depression may help both sets of symptoms [21]. However, if depression and fatigue remain distinct, then separate and more detailed assessment of both constructs could assist with more accurate diagnoses [13], and targeted interventions for depressive and fatigue symptoms may be warranted [22, 23].

### Current study

Unsettled infant behaviour occurs in ~ 25% of infants, and refers to persistent and inconsolable infant crying, resistance to soothing, short sleep intervals and frequent night awakenings [24]. Previous studies among women seeking support for unsettled infant behaviour have shown that many of these women experience elevated depression, anxiety, and fatigue symptoms [25–28]. Examining the profiles of these symptoms among women presenting at clinical services offering support for unsettled infant behaviour represents a unique opportunity to investigate whether depression and fatigue can be differentiated among women with elevated postpartum fatigue and psychological distress, and thereby address the previous lack of research in the relationship between depression and fatigue in clinical samples.

For this purpose, this study aimed to compare a one-factor model of combined depression and fatigue with a two-factor model of separate but related depression and fatigue. It was hypothesised that a two-factor model of related but separate depression and fatigue latent factors would provide a better fit for the data than a one-factor model, as is the case in community studies. To further demonstrate the predictive utility of the better fitting model, we explored the association(s) between the latent factor(s) and self-reported sleep efficiency given that sleep disturbance is related to both postpartum depression and fatigue [15, 29–33].

## Methods

### Study context and participants

Participants were women with infants aged up to 24 months who had been referred by medical practitioners to attend the Masada Private Hospital Early Parenting Centre (MPHEPC; Melbourne, Australia) for a residential early parenting program that assists with unsettled infant behaviour (for details on the intervention: [4, 24, 26, 34]). All women admitted to the MPHEPC between the 1st June 2015 and 12th October 2015 were invited to participate in the study with no exclusion criteria. Recruitment was carried out via advertisement on the MPHEPC website, a pamphlet in admission documentation, or by researchers on site. Participants completed a survey booklet on the first day of their admission before commencing treatment. The Avenue Hospital Research Ethics Committee (Trial 182) and Monash University Human Research Ethics Committee (CF15/1233) provided ethical approval. Written informed consent was obtained from all participants.

### Procedure

On the day of arrival to the MPHEPC, participants that expressed interest in the research project underwent an informed consent process and provided with a survey booklet that included the measures in this study. The survey booklet was returned to the researchers on site.

### Measures

#### Demographics

Maternal and infant demographics were collected through self-report and medical records extraction (see Table 1).

**Table 1:** Maternal and infant demographics (*N* = 167)

Demographic Variable	*n*	%
Country of Birth:		
Australia	117	70.09
Other	48	28.74
Language spoken at home:		
Mainly English	146	87.43
English & other	18	10.78
Mainly other	3	1.80
Mental Health History:		
Previous treatment^a^	58	34.73
No previous treatment	109	65.27
Relationship status:		
Married	144	86.23
De facto (living together)	21	12.57
Separated	1	0.60
Single	1	0.60
Education Level:		
University or higher university degree	129	77.25
Certificate/diploma/trade	29	17.97
Completed secondary school	4	2.40
Partial completion secondary school	1	0.60
Multiple Birth:		
Single birth	162	97.01
Twins	5	2.99
Parity:		
Primapara	83	49.70
Multipara	58	34.73
Infant Health:		
Excellent	84	50.30
Very Good	70	41.92
Good	9	5.39
Fair	2	1.20

*Note*. ^a^ Among the 58 who reported having received previous mental health treatment, 36 received treatment for depression, 38 for anxiety, and 5 for posttraumatic stress disorder

#### Depression

The Depression Anxiety Stress Scales Depression subscale (DASS21-D) [35] is a widely used 7-item measure of depressive symptoms during the last week. The DASS21-D has adequate validity and reliability for postpartum populations [11, 36]. For this study Cronbach’s alpha was .88, Omega was 0.89, and Greatest Lower Bound was 0.92 [37].

#### Fatigue

A revised five-item version of the Fatigue Severity Scale (FSS; [38]) was used to measure the interference of fatigue on functioning. The FSS is a widely used scale of fatigue severity and interference in chronic illness populations. Similar to findings in other chronic illness populations [39–42], the full nine-item FSS had several psychometric issues based on Rasch analysis [43]. The revised version (FSS-5R) was calculated from Items 4 to 8 of the original FSS with simplified response options (recoded from 1,234,567 to 1,112,345) and had improved psychometric properties [43]. Scale items are listed in Additional file 1: Table S1. For the FSS-5R, Cronbach’s alpha was .87, Omega was .88, and Greatest Lower Bound was .89. Scores on the full FSS-9 were also used to calculate the proportion of women reporting fatigue severity above the suggested clinical cut-off (≥ 36) and for comparison with community studies in which the full scale was used.

### Sleep quality

Sleep Efficiency (SE) represents overall sleep quality, and was calculated as the percentage of self-report total sleep time against time spent in bed over the past week. SE ranges from 0% (low) to 100% (high efficiency).

The following well-validated instruments were also used to characterise the overall psychological distress reported by the sample: Depression Anxiety Stress Scale Anxiety (DASS21-A) and Stress (DASS21-S) subscales [35]; Insomnia Severity Index (ISI; [44]); and the 6 item version of the Irritability Depression Anxiety Scale – Irritability subscale (IDA-I; [45]).

### Data analysis

**Fig. 1:**
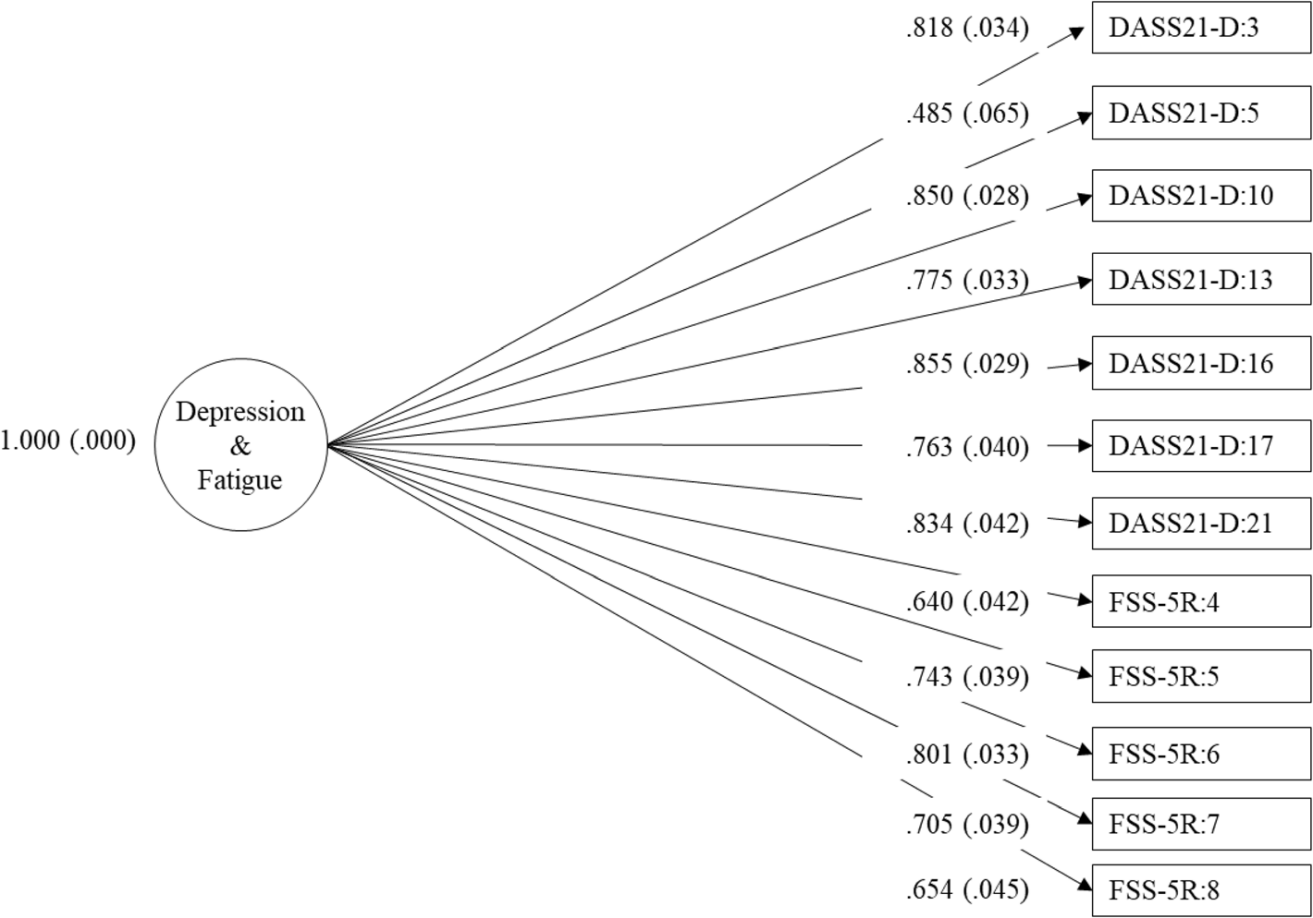
One-factor model of depression and fatigue. Note: Loadings are standardised

**Fig. 2:**
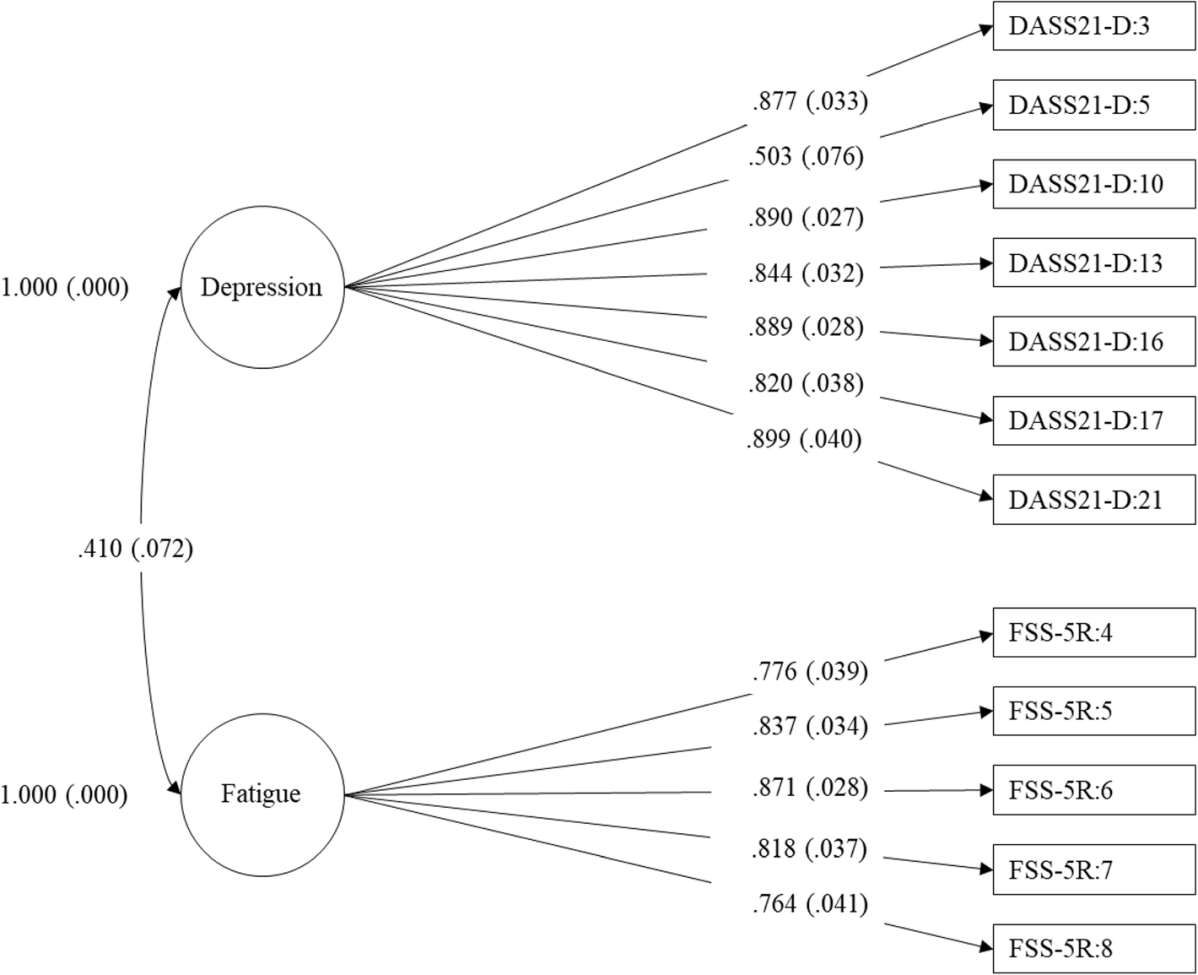
Two-factor model of depression and fatigue*.* Note: Loadings are standardised

Data analysis was conducted in Mplus Version 7.4 [46]. First, one-factor models of depression using the DASS21-D and fatigue using the FSS-5R were assessed separately to confirm the uni-dimensionality of each scale. Then, one- and two-factor models for depression and fatigue were conducted and compared. In the one-factor model, all depression and fatigue items loaded onto a single latent variable representing a single combined construct (see Fig. 1 below). In the two-factor model, items from the DASS21-D and the FSS-5R were separately loaded onto their respective latent variables; the depression and fatigue latent variables were allowed to be correlated (see Fig. 2). Thus, the two-factor model tests whether depression and fatigue are separate but correlated constructs [13].

Confirmatory factor analysis (CFA) analysis was conducted using diagonally weighted least squares (WLSMV) estimation [47]. The sample size (*N* = 167) had power of 0.80 to identify an effect size of 0.30 [48] and exceeded 10 observations per parameter [49]. The criteria for adequate model fit were: Chi-Square Test of Model Fit, Root Mean Square Error of Approximation (RMSEA) ≤ 0.05, Comparative Fit Index (CFI) and Tucker-Lewis Index (TLI) > 0.9, and Weighted Root Mean Square Residual (WRMR) < 1.0 [50, 51]. Comparison of model fit was carried out using the Chi-Square difference test for WLSMV estimation. Discriminant validity of the two-factor model was also assessed by examining the standardised pattern and structure coefficients of the two-factor model of depression and fatigue [13, 52]. Discriminant validity is established if the difference in values of the pattern and structure coefficients is .2 or above [13]. Finally, the predictive utility of the better fitting model was assessed by adding SE as the predictor of the latent factor(s). As missing data were low (< 5%), they were handled using pairwise deletion. No model modifications were made.

## Results

During the 19-week recruitment period, 167 of the 380 women admitted to the MPHEPC (44%) completed the study. Maternal and infant demographics and descriptive statistics for the DASS21-D, FSS-5R and SE are reported in Table 1 and Table 2. Missing data were minimal: 1.1% for the DASS21-D, 0.3% for the FSS-5R, and 4.8% for SE. A correlation matrix of scale items is in Additional file 1: Table S2. Participants reported elevated depressive symptoms, with 50% reporting symptoms at or above the published cut off for mild depressive symptoms (DASS21-D ≥ 5). Fatigue symptoms were also elevated, with 87% of women reporting fatigue severity above the suggested clinical cut-off (≥ 36) for the full FSS-9; scores were higher than those reported in a postpartum community population [22]. Scores on the other measures also point to an overall elevated level of distress in this sample. Forty-eight percent of women reported at least mild anxiety (DASS21-A ≥ 4), 64% reported at least mild stress (DASS21-S ≥ 8), and 46% reported insomnia symptoms in the clinical range (ISI ≥ 15).

**Table 2:** Descriptive statistics (*N* = 167)

Variable	*n*	*M*	*SD*	Median	Min.	Max.
Maternal Age (years)	167	34.26	4.23	34.00	24.00	49.00
Infant Age (months)	167	8.51	4.16	7.50	2.00	23.50
FSS-9	163	47.92	8.85	49.00	21.00	63.00
FSS-5R	163	17.35	5.24	18.00	5.00	25.00
DASS21-Depression	162	5.12	3.81	5.00	0.00	18.00
DASS21-Anxiety	162	3.59	2.90	3.00	0.00	13.00
DASS21-Stress	158	9.71	4.11	9.00	0.00	20.00
IDA-I	165	7.28	3.92	7.00	1.00	18.00
ISI	165	13.76	5.13	14.00	1.00	28.00
Sleep Efficiency (%)	159	62.94	15.79	63.16	23.08	100.0

*Note. DASS21-D* Depression Anxiety Stress Scale, *FSS-9* Fatigue Severity Scale, *FSS-5R* Fatigue Severity Scale Revised 5-item version, *IDA-I* Irritability Depression Anxiety Scale – Irritability subscale 6 item version, *ISI* Insomnia Severity Index

### Confirmatory factor analysis

Separate models of the DASS21-D and FSS-5R both showed acceptable fit without modification (see Table 3). For both models, the standardised coefficients all significantly loaded onto the latent factor (all *p*-values < .001) and all exceeded .78 (see Table 4), except for DASS21-D Item 5. Therefore, both scales uni-dimensionally assessed the respective constructs.

**Table 3:** Fit Indices for models (*N* = 167)

Model	*χ* ^2^	*df*	*p*	RMSEA [90% CI]	TLI	CFI	WRMR
Congeneric DASS21-D	25.77	14	.0277	0.071 [0.023, 0.113]	0.992	0.995	0.583
Congeneric FSS-5R	14.38	5	.0134	0.106 [0.044, 0.172]	0.984	0.992	0.441
One-factor model	475.56	54	<.0001	0.216 [0.199, 0.234]	0.812	0.847	2.410
Two-factor model	136.28	53	<.0001	0.097 [0.077, 0.117]	0.962	0.970	0.987
Two-factor model on SE	147.72	63	<.0001	0.092 [0.073, 0.111]	0.956	0.964	0.965

*Note*. *χ*^2^ Chi-Square Test of Model Fit; *CFI* Comparative Fit Index, *DASS21-D* Depression Anxiety Stress Scale Depression subscale, *FSS-5R* Fatigue Severity Scale-Revised 5-item version, *RMSEA* Root Mean Square Error of Approximation, *SE* Sleep Efficiency, *TLI* Tucker-Lewis Index, *WRMR* Weighted Root Mean Square Residual

**Table 4:** Pattern and structure standardised coefficients DASS21-D and FSS-5R

Scale and Item	Congeneric model	One-factor model	Two-factor model^a^			
			Depression		Fatigue	
	Pattern	Pattern	Pattern	Structure	Pattern	Structure
DASS21-D						
Item 3	0.89	0.82	0.88	0.88	0.00^b^	0.36
Item 5	0.39	0.49	0.50	0.50	0.00^b^	0.21
Item 10	0.90	0.85	0.89	0.89	0.00^b^	0.36
Item 13	0.83	0.78	0.84	0.84	0.00^b^	0.35
Item 16	0.89	0.86	0.89	0.89	0.00^b^	0.36
Item 17	0.84	0.76	0.82	0.82	0.00^b^	0.34
Item 21	0.90	0.83	0.90	0.90	0.00^b^	0.37
FSS-5R						
Item 4	0.79	0.64	0.00^b^	0.32	0.78	0.78
Item 5	0.83	0.74	0.00^b^	0.34	0.84	0.84
Item 6	0.86	0.80	0.00^b^	0.36	0.87	0.87
Item 7	0.81	0.71	0.00^b^	0.34	0.82	0.82
Item 8	0.78	0.65	0.00^b^	0.31	0.76	0.76

*Note*: ^a^ In the two-factor model all correlations were free to be estimated, and factor variances were set to unity for model identification. ^b^ Parameters were fixed at 0.00. *DASS21-D* Depression Anxiety Stress Scale Depression subscale, *FSS-5R* Fatigue Severity Scale-Revised 5-item version; Values are standardised coefficients

The one-factor model with depression and fatigue items loading onto a single construct had a poor fit (see Table 3). All items loaded significantly on the single latent factor (*p* < .001) and the standardised coefficients ranged from 0.49 to 0.86 (see Table 4 and Fig. 1). The two-factor model of depression and fatigue as related but separate latent factors provided an acceptable and improved fit (see Table 3). The standardised coefficients for fatigue items on the fatigue latent factor and depression items on the depression latent factor were all significant (*p* < .001) (see Table 4 and Fig. 2). Compared to the one-factor model, the two-factor model provided a significantly better fit to the data, Δχ^2^ (1) = 67.50, *p* < .001. The correlation between the fatigue and depression latent factors in the two-factor model was 0.41 (*p* < .001).

The pattern and structure coefficients of the one and two-factor models are shown in Table 4. The differences between the structure and the fixed pattern coefficients ranged from 0.21 to 0.39 for both the depression and fatigue items, suggesting adequate discriminant validity.

In the better fitting two-factor model, SE was added as a simultaneous predictor of both the depression and fatigue latent factors. This model had an acceptable fit to the data without modification (see Table 3). Lower SE was associated with both higher depression (*p* = .004) and fatigue (*p* < .001), with no significant difference in the strength of these two paths, Wald χ^2^ (1) = 0.131, *p* = .71.

## Discussion

In this sample of women with elevated psychological distress and fatigue symptoms seeking support for unsettled infant behaviour, depression and fatigue symptoms are best considered as separate constructs that share a moderate correlation. Furthermore, both constructs were simultaneously predicted by a potential common cause (i.e., sleep efficiency), suggesting that the two-factor model may facilitate the understanding of the risk factors for both conditions. This study also supports the DASS21-D and a revised FSS-5R as uni-dimensional measures of depressive and fatigue symptoms in this population.

Findings from this study echo results from community postpartum populations where depression and fatigue were also found to be separate constructs [11, 13]. However, the correlation between the depression and fatigue latent factors in this study was smaller than the large associations seen in the two studies that applied CFA on non-clinical samples [11, 13]. This could be because in this study, depression levels while elevated are not severe based on cut-off scores, while fatigue levels are high based on cut-off scores, thus the difference between the two constructs may be more prominent. Alternatively, the lower correlation in this study could be due to differences in scales: the DASS21-D does not include any fatigue or somatic items, and the FSS-5R assesses fatigue interference rather than specific symptoms. This combination may have led to a weaker correlation between the two factors in this study compared to other combinations of scales. Nevertheless, the correlation between depression and fatigue in this study is comparable to that in other postpartum studies (*r* = .30 to .45; [10, 53–58]).

Our analyses on sleep efficiency serve as an example of many potential uses of the two-factor model in understanding common predictors and mechanisms. In this study, the findings were consistent with the literature linking self-report sleep disturbance with both postpartum fatigue and depressive symptoms [14, 15, 29–33].

### Limitations and strengths

As participants in this study were predominantly university-educated, born in Australia, and had the necessary resources to access privately funded treatment, this may limit the generalizability of our results. Also, despite overall high distress levels, depressive symptoms reported in our study were not severe. Thus, findings may not generalise to mothers meeting diagnostic criteria for a depressive disorder. A further limitation was that our sample comes from an ongoing clinical service that admits infants of 0–2 years, and infants in this study had an age range spanning 21.5 months. During this period, various psychological, biological and social factors may influence depression and fatigue. It is also possible that our sample could have included women with chronic health difficulties that contribute to their reported symptoms. Finally, given that the service we recruited from only admitted women with their infants, this paper did not examine the how potentially elevated mental health symptoms in partners [59] impact women’s experiences and symptoms.

Nevertheless, this study represented a unique opportunity to investigate the relationship between depression and fatigue in a clinical postpartum population with elevated psychological distress and fatigue symptoms. Given the high prevalence of infant settling difficulties in the community, these results are likely to be relevant to a high proportion of women who have given birth in the last year or two [24]. Other strengths include a large sample size, a relatively high recruitment rate for a help-seeking population (44%), and the use of appropriate statistical modelling. A further strength of this study was that it serves as a demonstration of how a third construct such as sleep efficiency can influence both these constructs.

## Implications and conclusions

Theoretically, our findings add further support for the two-factor model of related but distinct postpartum depression and fatigue and show that depression and fatigue likely remain distinct constructs, even when mothers are experiencing elevated psychological distress and fatigue levels. By showing how sleep efficiency can be independently related to both the depression and fatigue factors, this study demonstrated the potential utility of the two-factor model for understanding other potential physiological, psychological, and situational factors that could underlie both conditions [12, 60].

Clinically, our results indicate that among women seeking support for unsettled infant behaviour, and perhaps more broadly, women who present to clinical services with high psychological distress and fatigue in the postpartum period, depression and fatigue symptoms need to be assessed and treated in their own right. Improved assessment and greater awareness that depression and fatigue are related but separate constructs could help prevent the diagnosis of fatigue symptoms as depressive symptoms [13, 14]. Given that fatigue is one of the DSM-5 diagnostic criteria for Major Depressive Disorder [5], some overlap between these two constructs is inevitable. However, more detailed assessment of both conditions will assist clinicians to determine whether impaired postpartum functioning is caused by depressive symptoms, fatigue symptoms, or a combination of both.

Women experiencing fatigue but not depression may benefit from targeted interventions for fatigue, rather than potentially more intensive pharmacological treatments or therapy that may be better suited for depression [13, 22, 23]. Residential early parenting programs that assist with unsettled infant behaviour have demonstrated efficacy in rapidly reducing fatigue and may be an appropriate treatment in this situation [27, 34].

## Additional file

Additional file 1: **Table S1.** Summary of Items for FSS-5R and DASS21-D. **Table S2.** Means, standard deviations, and Pearson correlations for FSS-5R, DASS21-D and SE (*N* = 167). (DOCX 41 kb)

## Abbreviations

CFA: Confirmatory Factor Analysis
CFI: Comparative Fit Index (CFI)
DASS21: Depression Anxiety Stress Scale
DSM-5: Diagnostic and Statistical Manual of Mental Disorders, 5th Edition
FSS-5R: Fatigue Severity Scale - Revised 5 item version
FSS-9: Fatigue Severity Scale
IDA-I: Irritability Depression Anxiety Scale – Irritability subscale, 6 item version
ISI: Insomnia Severity Index
MPHEPC: Masada Private Hospital Early Parenting Centre
RMSEA: Root Mean Square Error of Approximation
SE: Sleep Efficiency
TLI: Tucker-Lewis Index
WLSMV: Diagonally weighted least squares estimation
WRMR: Weighted Root Mean Square Residual
