# Supplementary material for: Related but different: distinguishing postpartum depression and fatigue among women seeking help for unsettled infant behaviours
Source: BMC Psychiatry. 2018 Sep 25;18:309. doi: 10.1186/s12888-018-1892-7 (PMC6156854; doi:10.1186/s12888-018-1892-7)
Supplement: Supplementary file 1 — Table S1. Summary of Items for FSS-5R and DASS21-D. Table S2. Means, standard deviations, and Pearson correlations for FSS-5R, DASS21-D and SE (N = 167). (DOCX 41 kb) [file 12888_2018_1892_MOESM1_ESM.docx]

Supplement

Table S1

Summary of Items for FSS-5R and DASS21-D

| *DASS-21* | | *FSS-5R* | |
| --- | --- | --- | --- |
| DASS21-D:3 | Lack positive feeling | FSS-5R:4 | Interference with physical functioning |
| DASS21-D:5 | Lack initiative | FSS-5R:5 | Frequency of fatigue problems |
| DASS21-D:10 | Nothing to look forward… | FSS-5R:6 | Prevention of sustained physical functioning |
| DASS21-D:13 | Down-hearted | FSS-5R:7 | Interference with responsibilities |
| DASS21-D:16 | Lack enthusiasm | FSS-5R:8 | Fatigue among most disabling symptoms |
| DASS21-D:17 | Worthlessness |  |  |
| DASS21D:21 | Meaningless |  |  |

*Note.* DASS21-D: Depression Anxiety Stress Scale Depression subscale; FSS-5R: Fatigue Severity Scale-Revised 5-item version.

Table S2

Means, standard deviations, and Pearson correlations for FSS-5R, DASS21-D and SE (*N* = 167)

| Variable | *M* | *SD* | 1 | 2 | 3 | 4 | 5 | 6 | 7 | 8 | 9 | 10 | 11 | 12 |
| --- | --- | --- | --- | --- | --- | --- | --- | --- | --- | --- | --- | --- | --- | --- |
| 1. FSS-5R:4 | 3.79 | 1.19 |  |  |  |  |  |  |  |  |  |  |  |  |
| 2. FSS-5R:5 | 3.15 | 1.34 | .63** |  |  |  |  |  |  |  |  |  |  |  |
| 3. FSS-5R:6 | 3.10 | 1.35 | .60** | .67** |  |  |  |  |  |  |  |  |  |  |
| 4. FSS-5R:7 | 3.41 | 1.25 | .54** | .56** | .62** |  |  |  |  |  |  |  |  |  |
| 5. FSS-5R:8 | 3.88 | 1.26 | .49** | .52** | .58** | .61** |  |  |  |  |  |  |  |  |
| 6. DASS21-D:3 | 0.73 | 0.74 | .11 | .26** | .26** | .25** | .09 |  |  |  |  |  |  |  |
| 7. DASS21-D:5 | 1.31 | 0.69 | .28** | .24** | .35** | .42** | .33** | .31** |  |  |  |  |  |  |
| 8. DASS21-D:10 | 0.61 | 0.72 | .16* | .27** | .22** | .20* | .13 | .68** | .24** |  |  |  |  |  |
| 9. DASS21-D:13 | 0.96 | 0.80 | .21** | .31** | .33** | .28** | .21** | .64** | .31** | .60** |  |  |  |  |
| 10. DASS21-D:16 | 0.73 | 0.77 | .16* | .29** | .24** | .27** | .18* | .70** | .32** | .70** | .66** |  |  |  |
| 11. DASS21-D:17 | 0.51 | 0.74 | .09 | .19* | .18* | .14 | .05 | .60** | .15 | .60** | .57** | .56** |  |  |
| 12. DASS21D:21 | 0.24 | 0.52 | .17* | .18* | .16* | .16* | .08 | .52** | .15* | .64** | .51** | .52** | .68** |  |
| 13. SE | 62.94 | 15.79 | -.19* | -.27** | -.21** | -.22** | -.23** | -.22** | -.17* | -.15 | -.28** | -.19* | -.08 | -.10 |

*Note*. DASS21-D: Depression Anxiety Stress Scale Depression subscale; FSS-5R: Fatigue Severity Scale-Revised 5-item version; SE: Sleep Efficiency. * indicates p < .05; ** indicates p < .01.
